# Supplementary figures and images for: Natural variation in a glucuronosyltransferase modulates propionate sensitivity in a C. elegans propionic acidemia model
Source: PLoS Genet. 2020 Aug 28;16(8):e1008984. doi: 10.1371/journal.pgen.1008984 (PMC7482840; doi:10.1371/journal.pgen.1008984)

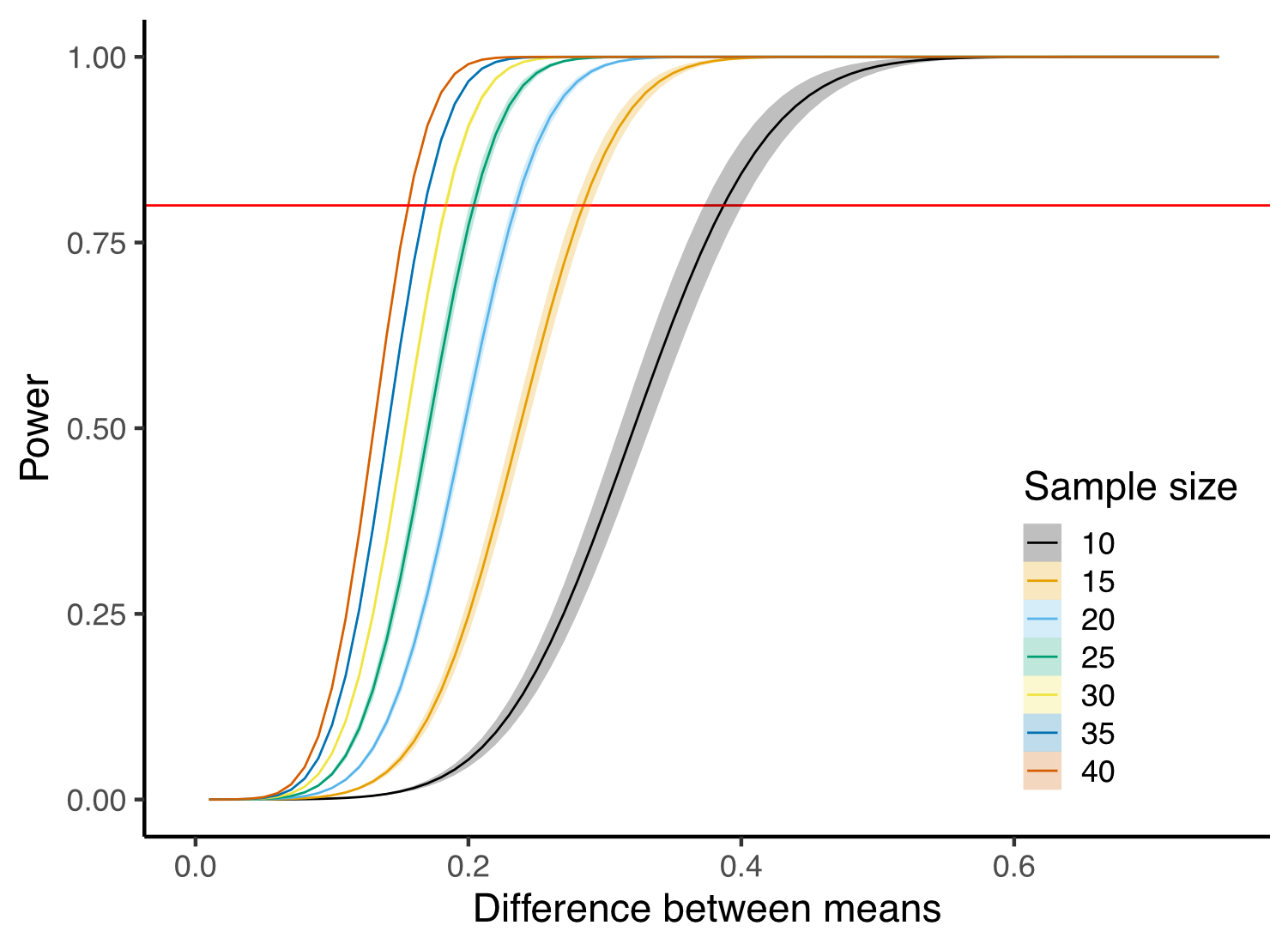

Supplement: S1 Fig — Power analysis of L1 survival after propionate exposure is shown. We calculated power for a range of mean differences from 0 to 1, using the average standard deviation of 100 subsamples from a large-scale experiment that measured DL238 propionate survival. The solid line represents the mean of 10 replicate power calculations and the shaded area around the solid lines represent the standard deviation of the replicates. The line colors represent the sample size. The dashed red line indicates 0.8 power. (TIFF) [file pgen.1008984.s001.tiff]

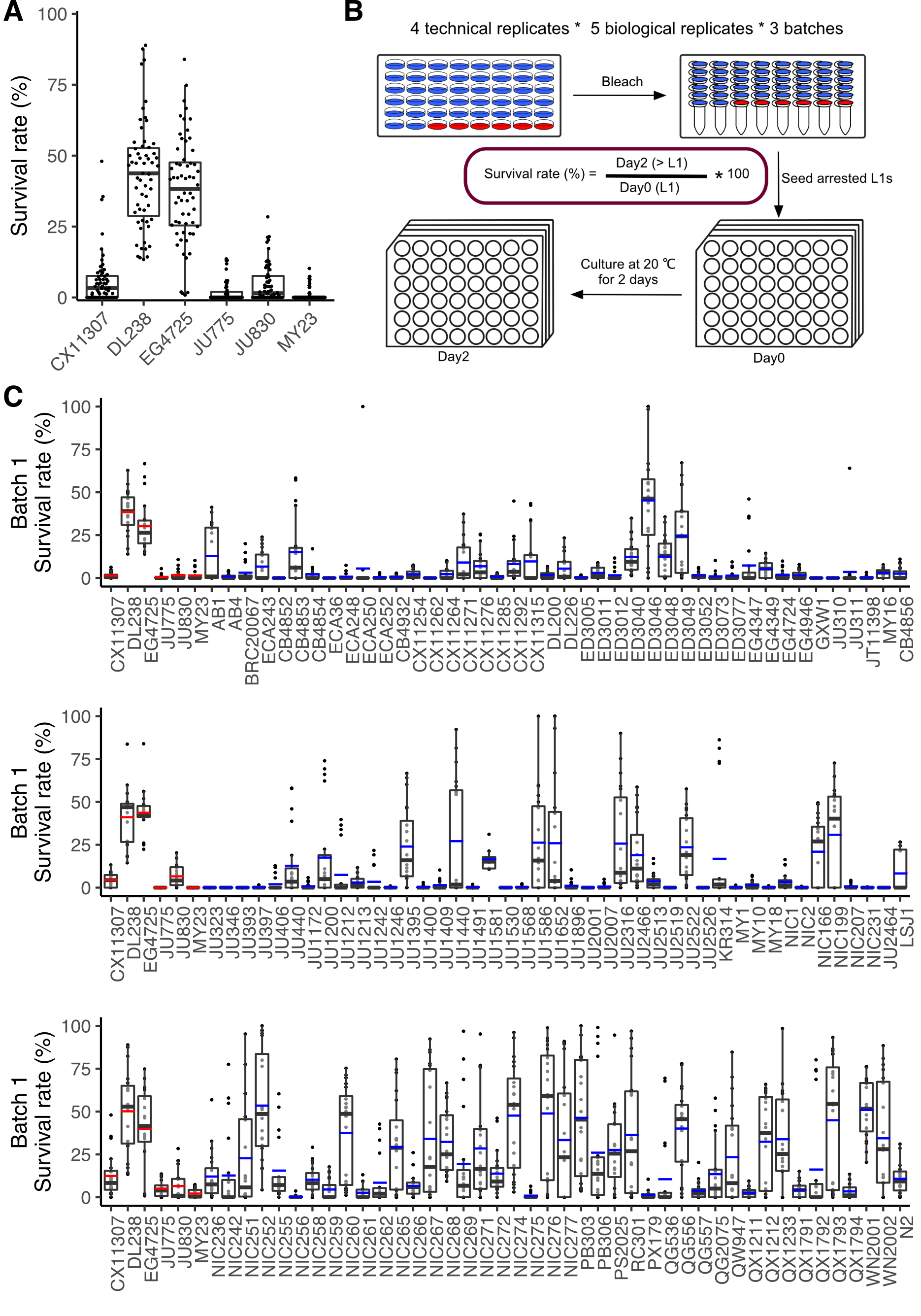

Supplement: S2 Fig — (A) L1 survival in the presence of 100 mM propionate for six C. elegans strains with eight technical plus five biological replicates. (B) Experimental setup to phenotype wild isolates for GWA mapping. 133 wild C. elegans strains were divided into three batches to test their survival after exposure to 100 mM propionate. Each batch contains 48 strains, including six control strains that control for batch effects. (C) L1 survival rate in the presence of 100 mM propionate for each 48 strain batch described in B. The colored bars represent the mean L1 survival for each C. elegans strain. Tukey boxplots overlay the data. Batch-control strains are indicated by red median bars. (TIFF) [file pgen.1008984.s002.tiff]

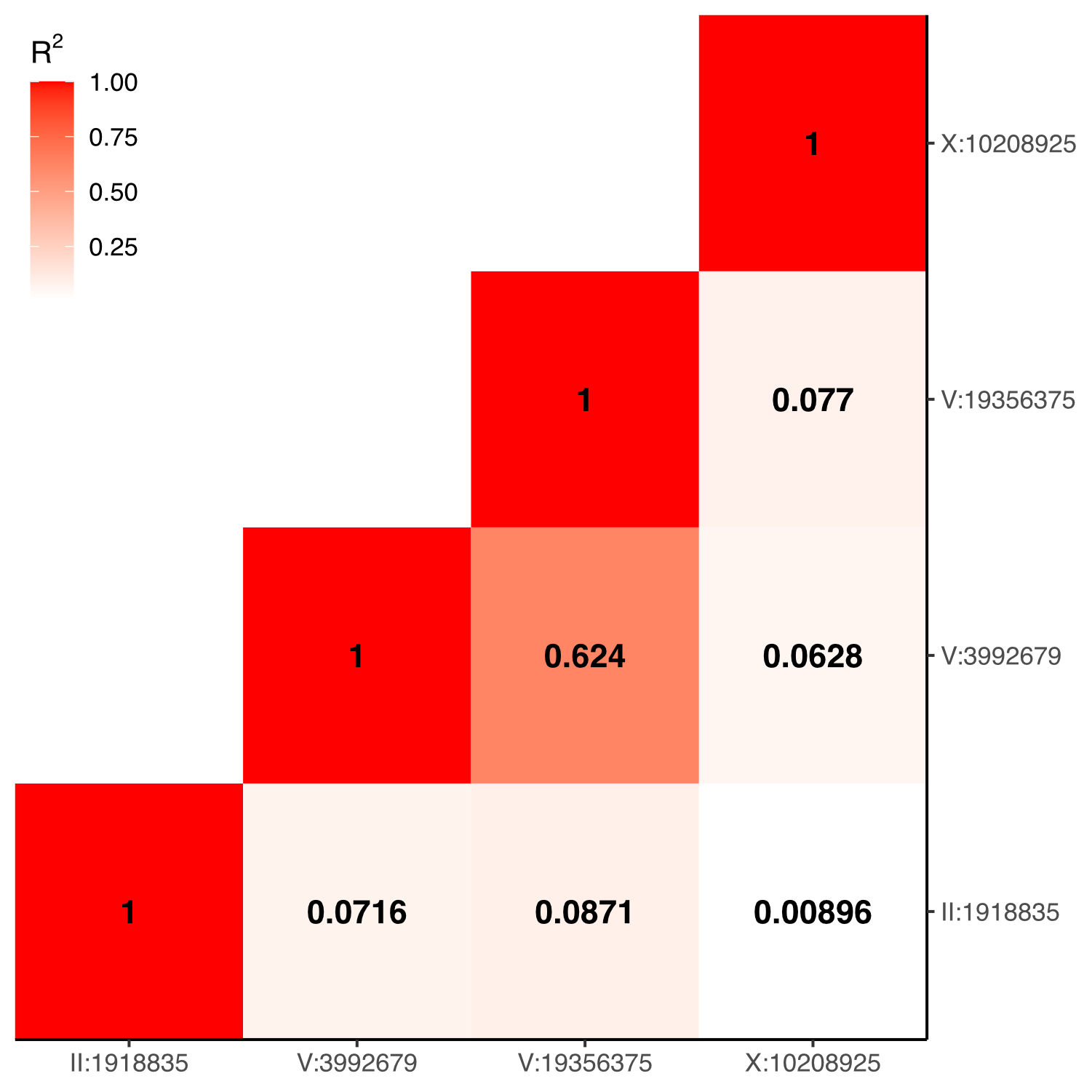

Supplement: S3 Fig — Linkage disequilibrium (r2) of peak QTL markers identified by genome-wide association mapping is shown. The tile color represents the correlation between marker pairs. (TIFF) [file pgen.1008984.s003.tiff]

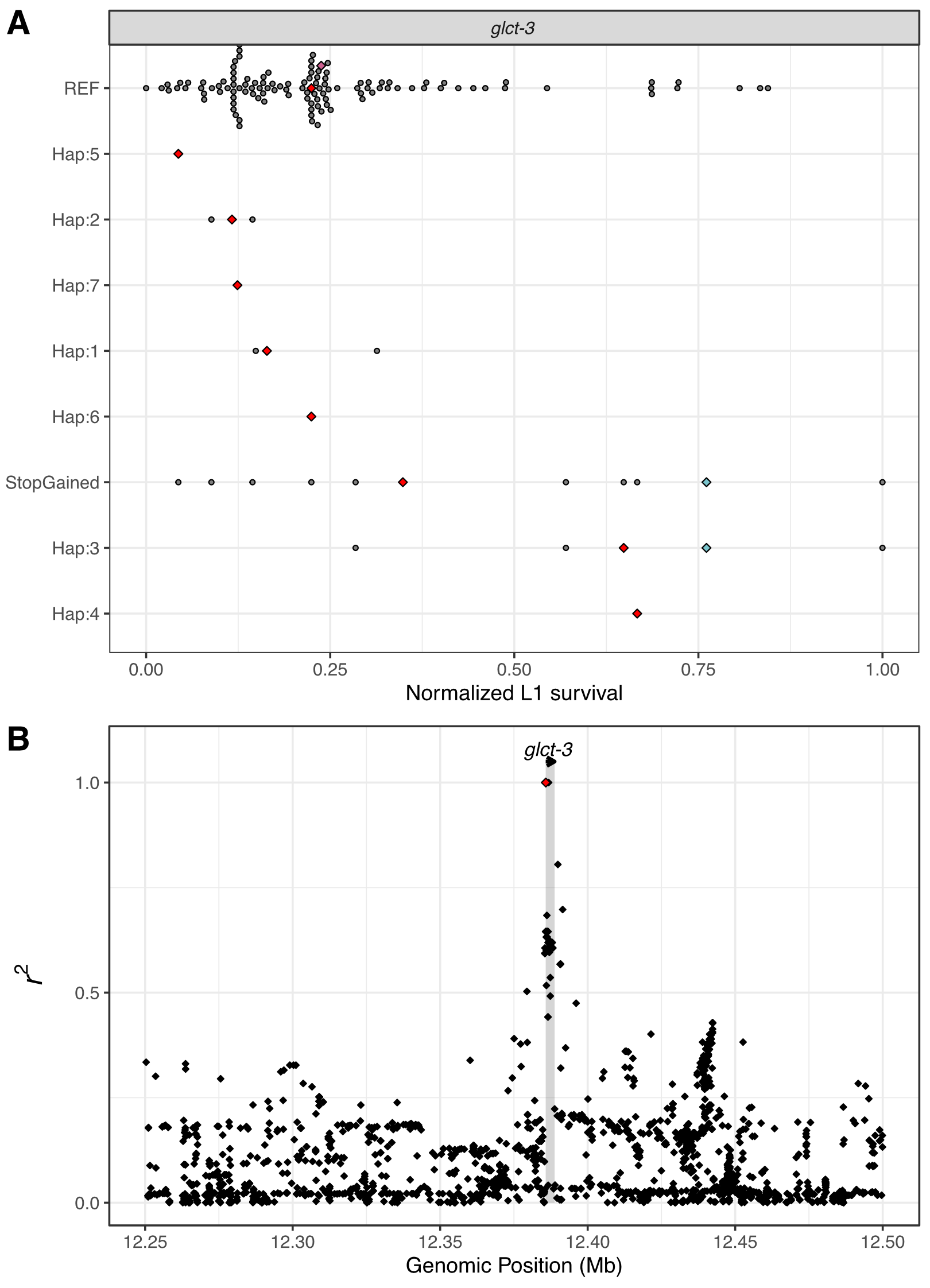

Supplement: S4 Fig — (A) The normalized L1 survival in the presence of propionate for each phenotyped C. elegans strain is shown on the x-axis. The y-axis represents unique haplotypes (numbered from 1:n) constructed from variants with moderate-to-severe predicted effects on glct-3 found to be significantly associated with propionate sensitivity. If a variant with a high predicted effect on gene function was identified, we plotted it separately. Therefore, a strain can be represented twice if it contains a variant with a high predicted effect on gene function. The red diamonds represent the median phenotype value for each unique haplotype. The blue and pink diamonds represent the DL238 and BRC20067 strains, respectively. (B) The pairwise linkage disequilibrium (r2) between the allele that encodes the Gly16* (red diamond) in GLCT-3 and all variants (black diamonds) in the surrounding genomic region is shown on the y-axis. The x-axis represents the genomic position (Mb) of each variant. (TIFF) [file pgen.1008984.s004.tiff]

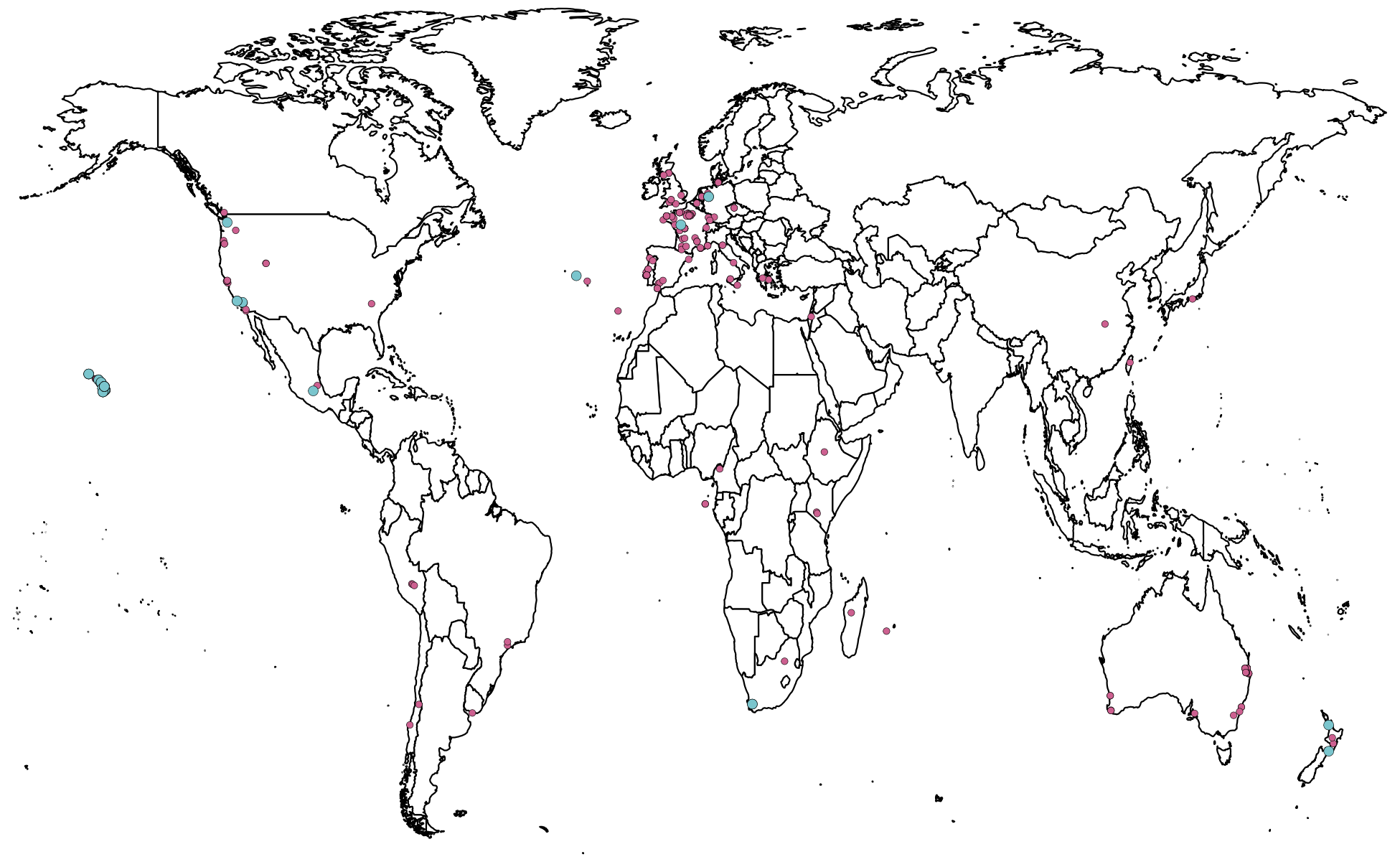

Supplement: S5 Fig — Sampling locations of wild C. elegans strains. Each dot represents the location where an individual strain was sampled. Pink dots represent strains carrying the REF allele at GLCT-3, and blue dots represent strains carrying the Gly16* allele. (TIFF) [file pgen.1008984.s005.tiff]

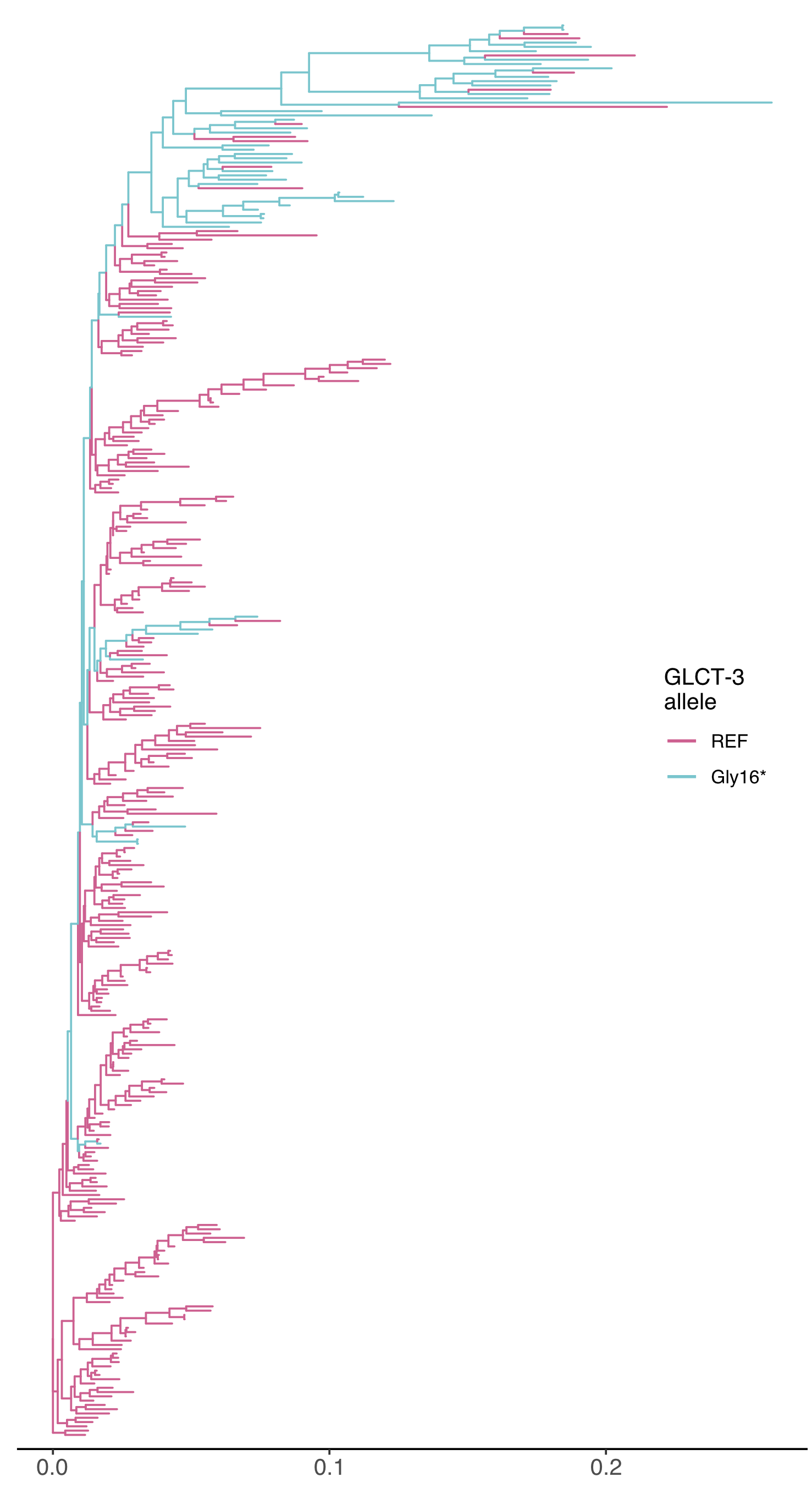

Supplement: S6 Fig — A maximum likelihood phylogenetic tree of the C. elegans population. Branches are colored based on the GLCT-3 allele the individual strain carries, blue represents strains with the GLCT-3 Gly16* allele, and pink represents strains with the reference allele. (TIFF) [file pgen.1008984.s006.tiff]

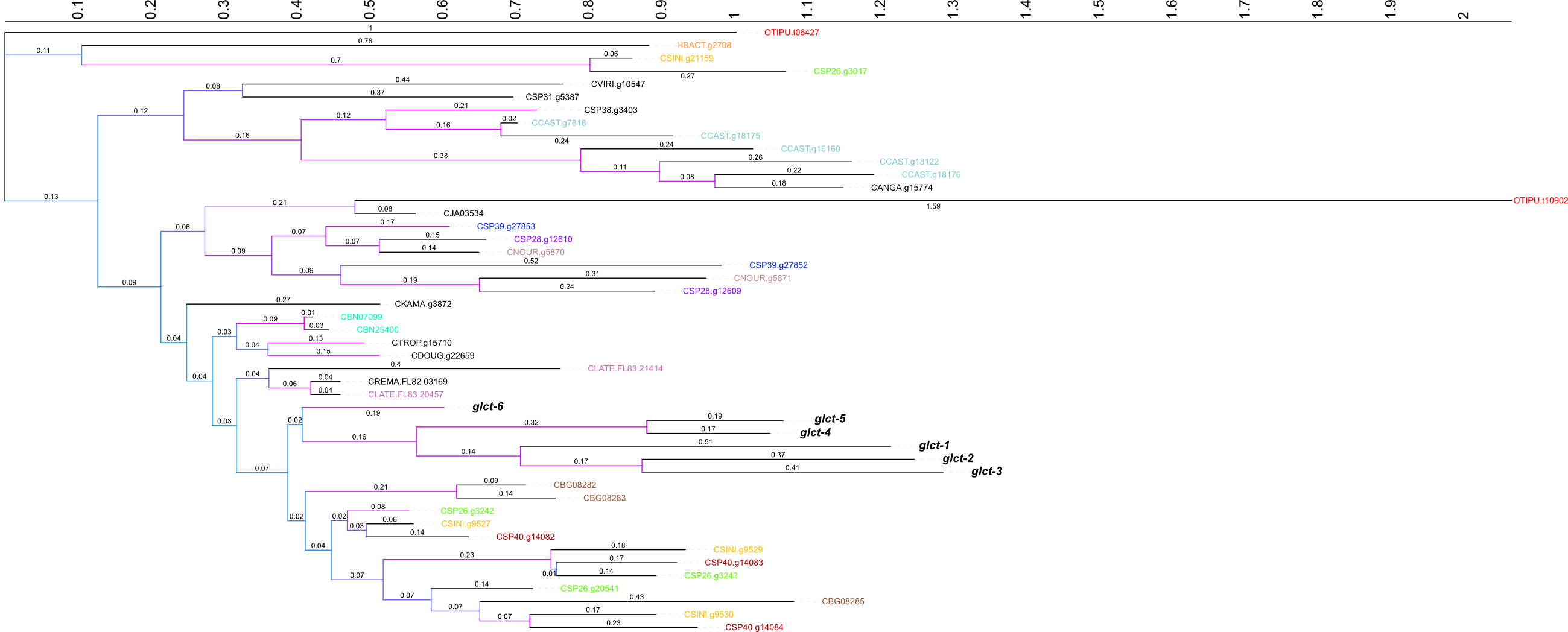

Supplement: S7 Fig — The maximum likelihood phylogenetic relationship of glct-3 homologs is shown. Branch lengths are shown above each branch. Branch colors correspond to the bootstrap support for the split, with pink indicating higher support. If a species contains more than homolog, all homologs for that species are colored the same color. Species with only one homolog are colored black. The C. elegans glct genes are colored in black and bolded. (TIFF) [file pgen.1008984.s007.tiff]

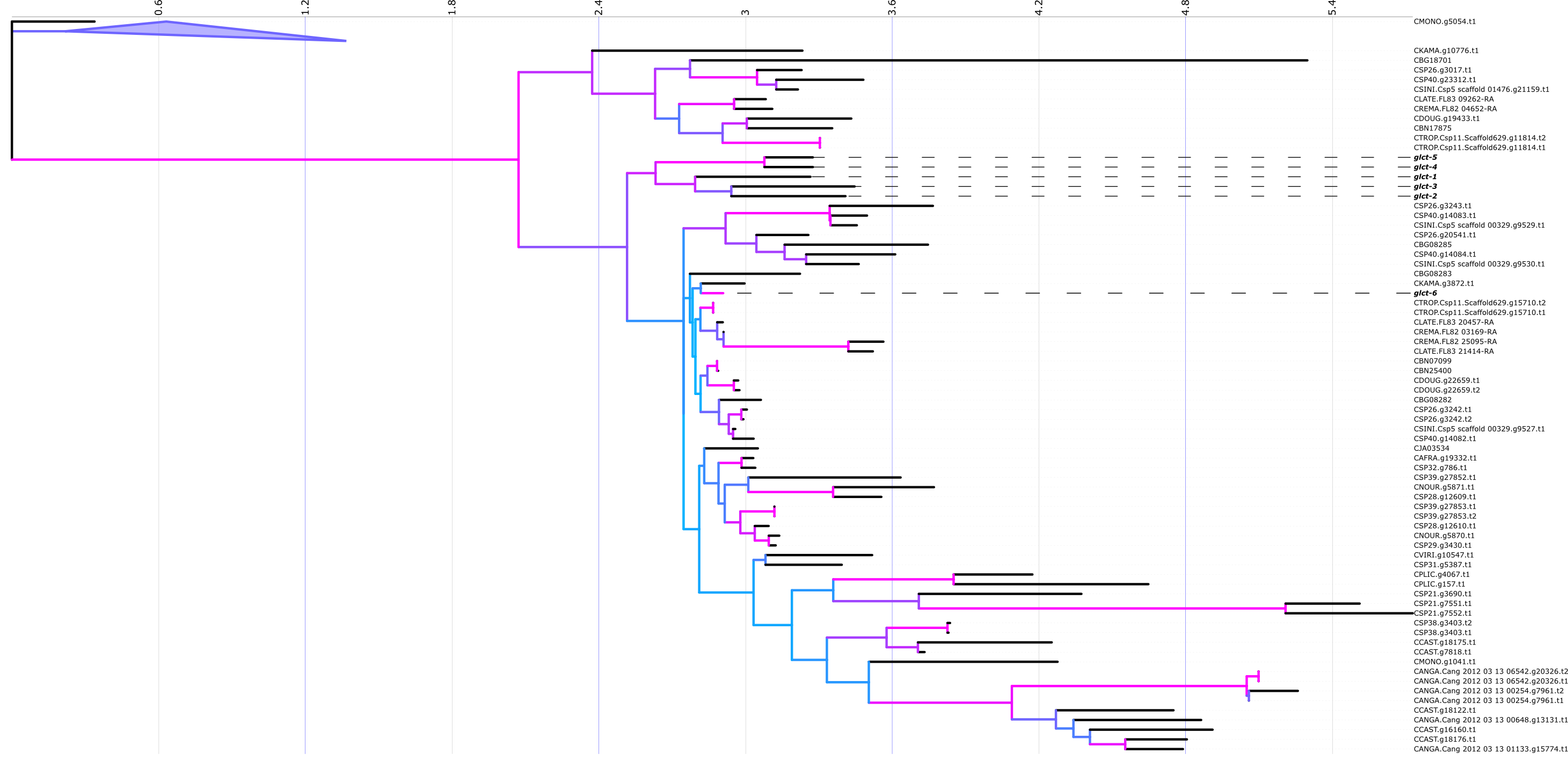

Supplement: S8 Fig — The maximum likelihood phylogenetic relationship of glct-3 homologs is shown. Branch colors correspond to the bootstrap support for the split, with pink indicating higher support. The C. elegans GLCT protein sequences are bolded. (TIFF) [file pgen.1008984.s008.tiff]
