# Supplementary material for: Natural variation in a glucuronosyltransferase modulates propionate sensitivity in a C. elegans propionic acidemia model
Source: PLoS Genet. 2020 Aug 28;16(8):e1008984. doi: 10.1371/journal.pgen.1008984 (PMC7482840; doi:10.1371/journal.pgen.1008984)
Supplement: Attachment — Submitted filename: 20200705_PropionateResponsesFinalSubmission.pdf [file pgen.1008984.s024.pdf]

Please find our responses below. We added a Methods section to accommodate the narrow-sense heritability estimates. Also, we edited the Discussion again. This gene class is distinct from the UGT enzymes, so we just discussed the GLCT gene family. Thank you for the comments!

Line 167: why is the heritability 0.53? From the figure I read something like 0.7 (also compared to the value in the previous version this is low).

On the draft in the email, 0.53 is on line 149. This estimate is different from the estimate in Figure 1, which is around 0.7 as the reviewer points out, because it was calculated based on the phenotypes from the GWA mapping experiment. Figure 1 heritabilities were estimated from the panel of strains used in the dose response experiment. This value is different from the previous version because we modified the outlier removal procedure when processing the GWA phenotypes as the reviewer recommended.

Line 260: the narrow-sense heritability is mentioned, but I cannot find the methods of how this was estimated.

On the draft in the email, the line in question is 242. We added this methods section at the end of the manuscript to explain how narrow-sense heritability was estimated:

Heritability estimates from genome-wide association phenotypes

We used the *sommer* package in R to calculate marker-based narrow-sense heritability [57]. We first calculated the marker-based heritability for the phenotype data used to perform GWA mapping using the *mmer* function in *sommer*, with the random variable in the model specified as *random=~vs(strain, Gu=A) + vs(strainE,Gu=E)*, where A is the additive genotype matrix (generated using the *A.mat* function in *sommer*) and E is the epistatic genotype matrix (generated using the *E.mat* function in *sommer*). To determine the effect of the GLCT-3 GLY16* allele, we performed the same heritability calculation, but we included the presence of the GLCT-3 GLY16* allele as a fixed effect in the *mmer* function. Narrow-sense heritability estimates were extracted from the *mmer* object using the *sommer pin* function with the formula *h2 ~ (V1) / (V1+V2+V3)*, where V1 is the additive variance component, V2 is the epistatic variance component, and V3 is residual variation. To determine the effect of the GLCT GLY16* allele, we performed the following calculation *(h2 - h2_glct3)/h2*, where *h2* is the narrow-sense heritability of the original phenotype data and *h2_glct3* is the estimate with the GLCT GLY16* allele as a fixed effect.

Line 320-326: I was not particularly helped by these statements in the discussion. Why do 50

references in human literature and a dozen references in C. elegans literature not provide enough ground for speculation? If current literature is not sufficient it would be more helpful to either discuss where the gaps are or options that are on/off the table. In other words, can you specify what makes it difficult to speculate? Because 'only' 50 or a dozen hits are not the reason this speculation is difficult.

On the draft in the email, the lines in question start at line 302. We modified the text to address the reviewers concerns. Of course, it is possible to speculate, and we have.
