# Supplementary material for: Natural variation in a glucuronosyltransferase modulates propionate sensitivity in a C. elegans propionic acidemia model
Source: PLoS Genet. 2020 Aug 28;16(8):e1008984. doi: 10.1371/journal.pgen.1008984 (PMC7482840; doi:10.1371/journal.pgen.1008984)
Supplement: Attachment — Submitted filename: 20200510_NA_ZDRALJEVIC_RESPONSES.docx [file pgen.1008984.s023.docx]

*We thank the three reviewers and editor for the insightful comments and corrections. Please find the* **reviewers’ comments in bold below***, and our responses in italics. We hope you agree that our manuscript is vastly improved and is now ready for publication in PLoS Genetics.*

**Editor overview:**

**This work is thoughtful and a useful demonstration of model system genetics and use of the CeNDR platform to conduct GWA inquiries. The reviewers in particular note that inclusion of the chrom V QTL is valuable, even though it turned out to be a false positive.**

**The main concern is about the larger biological context for this work. Reviewer 3 states that the major motivation for this paper is to understand human disease, but there is not much discussed on this point, nor on other studies seeking to identify modifier variants. Reviewer 1 is concerned about the interest/impact level of this work to the PLOS Genetics audience, and specifically mentions a low level of discussion around possible GLCT mechanisms. Like these reviewers, I also feel that the results in this manuscript don't quite meet the stated goal of the project--to use the tractable C. elegans model to identify variants/genes affecting propionate sensitivity, and thus gain insight into modifiers of human disease. (It would appear, based on references in the Intro, that the presence of modifier/genetic background effects for inborn errors in human metabolic disease is already well established. If so, the fact that this study illustrates functional variation in propionate sensitivity in natural populations is not especially impactful; but if that is not well established, perhaps an expanded discussion of the outstanding question(s) about variation in the human disease context would be valuable.) Given the idiosyncrasy of the C. elegans model, including GLCT gene family expansion, connecting even mechanistic findings in worms (which this work has not yet reached) to potential impact in human disease could still be hard, and still suffer from the same concern about relevance, so I fully acknowledge the challenge here. But, as written, it feels as though the potential biological insights that may be gleaned from this work have not been fully considered. Can the authors expand (more) upon the possible meaning behind showing that a putative glucuronosyltransferase affects propionate sensitivity? Only one paper is cited in the Discussion describing the function of glucuronosyltransferases. Is very little known about these enzymes?**

*We thank the editor for the comments. To be clear, genetic modifiers of rare metabolic disorders are extremely rare, in fact, almost none have been identified in humans, due to lack of power and because they are “Mendelian” disorders, for which even the idea of modifiers is only recently being appreciated. Thus, any modifiers of inborn errors of metabolism, identified in any model system are relevant data for the field.*

*Our study discovers a specific GLCT gene variant that can modify the “morbidity” of disease in a C. elegans model of propionic acidemia (i.e., worm phenotype). The role of glucuronosyltransferases in propionic acid sensitivity has not been observed previously, and not much is known about this enzyme family in metabolism. Our work puts forth a finding that human geneticists may consider investigating the role of GLCT pathways in this disease. We have added more detail throughout the manuscript to address this point, but we emphasize that the discovery of a specific gene variants that modifies a metabolic disorder is interesting to the community, especially because few to none of these connections exist.*

**Reviewers 2 and 3 both requested expanded explanations of methods, including better descriptions of the replication scheme and some specific requests for illustrating or reporting variation in the data. I also think Reviewer 2's points 6) and 7) are good ones and the ms will be well served by clarifying them. In addition to their comments below, I also was curious about how "GLCT function," generally interpreted, appears to associate with proprionate sensitivity. Of the six genes, GLCT-4 and GLCT-6 don't have much variation, but the other four do. Obviously strain DL238 has the stop-gain in GLCT-3 and the authors report that multiple strains have putative loss of function at two, three or four genes. What is DL238's status at the other GLCT genes? Of the wild isolates phenotyped, how many of them had putative loss of function at variable numbers of genes? Enough to ask (perhaps qualitatively) how loss of function across the GLCT family associates with proprionate sensitivity? Perhaps this was already explored, but I didn't see it mentioned in the ms. I'm curious about this because in my mind it goes to the biological context question raised above. If a more pointed hypothesis can be made about glucuronosyltransferase activity level and proprionate sensitivity, then the results speak to a potentially new biological insight, rather than a worm-specific, potentially even GLCT-3-specific, mechanism.**

*To answer your specific questions above, DL238 does not have any predicted amino acid-altering variants in any other glct family genes. We described in the Results the number of wild strains that have high impact coding variants in this family. Only variation in glct-3 is correlated with propionate sensitivity variation as shown by burden and marker-based mappings, so variation in the other family members do not significantly impact propionate sensitivity. In the plot below, you can see that only one wild strain has a variant in glct-1 and only a small number have variants in glct-6. The distribution of glct-6 does not support a role in this trait. The red point is the mean of the strains with glct variation; value is normalized propionate sensitivity.*

**Reviewers’ comments**

**Reviewer #1:**

**In this study, the authors used genome-wide association studies in C. elegans to identify genetic regulators of propionic acidemia. They identified one regulator, a gene encoding a putative glucuronosyltransferase. They generated a knockout allele of the gene, which confirmed its role in propionate toxicity. This work was carefully carried out, and a role for GLCT (B3GAT) in propionate metabolism is interesting, but puzzling.**

**At this point, with no mechanistic data or barely any speculation about how GLCT could be acting to render worms sensitive to propionate, it is hard to get excited about these results, and it does not seem like this work will generate general interest among readers.**

*We thank the reviewer for the comments. The Discussion contained speculation about the role of GLCT-3 in the presence of excess propionate. Unfortunately, our data point to an indirect modification mechanism, so follow-up mechanistic experiments would be difficult to define. We politely but strongly disagree that this study will not be of interest to general PLoS Genetics readers. As we mentioned above, few (if any) connections between metabolic disorders and genetic modifiers are known. This study offers one such connection. Overall, genetics is a discovery science, and the application of model organism genetics offers its most powerful application. Connections of natural differences across populations to any specific causal gene or variant are rare, so any results in this area should be emphasized for the lessons they can bring to C. elegans and beyond.*

**Reviewer #2:**

**Dear authors,**

**This paper presents a thorough set of experiments that lead to the identification of natural allelic variation in glct-3 linked to propionic acidemia in the model nematode Caenorhabditis elegans. The authors follow a clear experimental line: screening a limited set of wild isolates, screen a set of 133 wild isolates, follow-up by creation of introgression lines and a target-gene approach, after which the authors explore natural variation within and beyond the species for the glct gene family.**

**There are various interesting aspects in this study, including an interesting approach to the GWAS analysis (combining ‘classical’ GWA with burden-testing) and two-types of follow-up experiments. Here, I think, the authors should be applauded for including the results from the introgression line experiments, even though they did not lead to the identification of a gene or mechanism. Of course, I do have some comments and requests for clarification**

*We would like to thank the reviewer for appreciating the negative result that led us to the eventual discovery of glct-3. Very often, negative results are not reported and waste resources and time in future experiments. We thought it was important to relate these results for these reasons.*

**Major comments:**

**1. The authors do their best to explain how they conducted the experiments in replicates and how they dealt with the effects of having to split up the experiments in batches. However, it is not described what exactly constitutes a ‘technical’ and a ‘biological’ replicate, nor is it clear what exactly represents a ‘batch’. As the authors amass a large dataset on these factors, I would like the paper to include the actual effect of these levels of replications. These could be included in the materials and methods before the batch correction. An analysis of variance on these factors before and after batch correction would be good to assess the impact of this correction on the data.**

*We apologize for any confusion in the methods regarding replicates. We have modified the methods section to explain the following details:*

*A biological replicate is an independent growth of a strain, each done on a different day. For each biological replicate, strains were chunked from a starved plate, grown for two generations, bleached, synchronized as L1s in M9, and plated out on 48-well agar NGM plates with supplemented propionate, buffered with NaOH to maintain a pH of 6. For each technical replicate, approximately 100 arrested L1s were transferred to an individual well of a 48-well plate. Because we had a high level of replication, we were not able to complete the entire GWA phenotyping in one experiment and had to phenotype the strains in three separate sets. For each set, we included six reference strains as controls to verify that the batch conditions did not change significantly.*

*Regarding the effects of technical and biological replication and strain set, we performed analysis of variance as the reviewer suggested. To perform this analysis, we focused on the six strains that were included in all of the experiments. We found that the biological replicate (p-value = 0.026, Cohen’s F = 0.119) and set (p-value = 0.012, Cohen’s F = 0.134) had moderate, but significant effects on strain phenotypes. Furthermore, we found no significant effect of technical replicate (p-value = 0.83, Cohen’s F = 0.05), which is expected because technical replicates were drawn from the same strain preparation. As expected, these effects were all negligible after correction.*

**2. Next to presentation in the figures, can a table be included of both GWA analyses with the location of all variants above the threshold, the alt allele, the effect, and the variance explained?**

*Each QTL region has hundreds of variants above threshold, so we do not know the best way to display these data in the existing figure framework. We include these data as Supplemental Table 5 in the revised manuscript and trust this alleviates this concern.*

**3. In the discussion, in the last paragraph, the authors remark ‘that C. elegans is a fruitful “simple” model to identify genetic modifiers of inborn error in human metabolism’. While I fully agree with the idea that in C. elegans genetic and environmental control allow identification of pathway components that could easily have eluded human geneticists, the results presented here on the expansion of the glct gene family present another side of the coin. I would like to read the authors opinion on how a gene family expansion affects what we can infer from C. elegans as a model for human metabolic diseases. Is the canonical strain N2, as referred to in the introduction, the best genetic basis for studying propionic acidemia?**

*Over the evolution of a particular pathway, individual components have the potential to expand and contract via duplication or deletion. As these changes occur, novel connections can compensate for the altered copy number of a particular pathway component. It appears that we have identified such a novel link in the C. elegans species, where a loss of glct-3 function causes propionate resistance. Further characterization of the mechanism by which glct-3 contributes to increased propionate sensitivity has the potential to guide the search for modifiers of human propionic acidemia. We added some of these points to the Discussion.*

*We have previously used the N2 strain as a first model for propionic acidemia in worms. Specifically, we have used mutant animals, which harbor loss-of-function mutations in pcca-1, the ortholog of human PCCA. In humans, propionic acidemia is caused by loss-of-function mutations in both copies of either PCCA or PCCB mutations in humans. This defect leads to the toxic buildup of propionic acid (i.e., propionate) and other intermediate metabolites. C. elegans pcca-1 mutants show a similar metabolomic signature, such as elevated levels of 3-hydroxypropionate, and increased sensitivity to propionate.*

*Obviously, we cannot generate a true propionic acidemia model in each of the wild C. elegans isolates as it would require the generation of pcca-1 mutants in each background. Therefore, we instead opted to use each strain under low dietary vitamin B12 conditions, where flux through the canonical propionate breakdown pathway, which depends on vitamin B12, is low. As a result propionate accumulates and the animals are more sensitive to exogenously supplemented propionate (Watson et al., eLIFE 2016). This is discussed in the revised manuscript.*

**Minor comments (some have overlap with the major comments):**

**Line 84: ... toxicity, likely …**

*Thank you. We have updated the manuscript text.*

**Line 86-87: ‘The vast ... Bristol, England’. The point made here is very implicit and it would be good for readers to connect it more explicitly to the next sentence**

*We have updated the manuscript text.*

**Line 129-131: I think I’m clear on what ‘biological replicate’ means, however what constitutes a technical replicate is not described (neither in the M&M).**

*We have added the explanation of biological and technical replicates in the Materials and Methods section as described above.*

**Line 137: It seems this should be figure 1D?**

*We agree, and have changed the reference to Figure 1C, 1D. The reason that we keep referring to Figure 1C here is that EG4725 is also propionate resistant while hard to tell on Figure 1D because of the color.*

**Line 144-146: ‘To identify ... (H2= 0.79)’ Can you explain the rationale of subsampling for the detailed concentration range and not for the extended range?**

*We performed the subsampling procedure in the fine-scale experiment because the broad-sense heritability estimates were more similar. However, we repeated the subsampling procedure for the entire dose full dose-response experiment and obtained similar results (broad-sense heritability estimates are shown below for each concentration tested). The figure has been updated with this new analysis.*

**Line 154 – 156: ‘We tested ... effects (Figure S2)’. I got a bit confused here, is it correct the setup is that all 5 biological replicates with 4 technical replicates were done within the same batch? Together with my previous question on what constitutes a technical replicate this does not really clear things up. I currently read it as: we measured 20 ‘replicates’ of a strain at the same time and anchored these measurements with 6 strains included along all these measurements.**

*The biological replicates were five independent growths of each strain; each of the five independent growths of each strain have four technical replicates, which means each strain arrested L1s were seeded onto four different wells of 48-well plates, and the number of worms on each well was recorded individually as technical replicates. The six reference strains were considered as “new” strains for each batch, and the survival rate was scored separately.*

*A pilot experiment was carried out to test the six strain responses to propionate treatment. Forty data points, five independent biological replicates (eight technical replicates for each biological replicates) were scored for the six strains (Figure S2A), and because of the consistent performance upon propionate treatment, they were used as reference strains for the larger experiment.*

*We updated the Methods section to clarify.*

**Line 168: ‘Therefore, we ... gene basis [34]’. How many tests were conducted by SKAT; how many genes passed the thresholds set?**

*With the SKAT mapping, we identified one gene (glct-3) on chromosome I and 13 genes on chromosome V that were significantly associated with propionate responses. We have updated the mapping summary text in the Results to include these numbers.*

**Line 215: ‘this gene is major contributor’**

*We have made the change to ‘this gene is a major contributor’.*

**Line 222: why is Cohen’s F reported here, whereas in the figure a t-test is used**?

*We wanted to report the effect size corrected for the standard deviation in the data. However, we note that the effect size estimate from Cohen’s d is identical to the estimate from Cohen’s F in a comparison of two groups, and the estimates from a linear model are very similar to both Cohen’s d and F (29.5% for the BRC20067 deletion and 59.8% for the BRC20067 allele swap). We have changed the Figure description to Cohen’s F p-values as opposed to p-values from a t test.*

**Line 222-226: ‘The strains ... to propionate’. How does that compare to the effect size of the QTL detected?**

*The QTL effect size that corresponds to a loss of glct-3 function is approximately 10% of the total parental difference in the follow-up experiment. However, we note that the experimental setup for the glct-3 follow-up experiment was much simpler than the GWA mapping experiment, which likely contributes significantly to relatively small QTL effect relative to the parental difference. These details have been added to the manuscript in the discussion of the CRISPR follow-up experiment.*

**Line 340-342: ‘To process ... standard deviation’ how many data points were removed?**

*From a total of 715 data points (means of technical replicates), one of five data points was removed for each of 89 strains, two of five data points were removed from 11 strains, and three of 15 data points were removed from three strains (this last set of strains correspond to three of six of the strains present in each mapping set). We have updated the Methods section to include these numbers.*

**Line 345: ‘as above’; please describe how exactly, it is not clear of what you remove the outliers? How many values were removed?**

*Upon considering the reviewers question and discussion among the authors, we eliminated the second outlier removal step that previously occurred after correcting for biological replicate and strain set. We have updated the methods section to reflect this change in phenotype processing. We note that this change did not affect any of the major conclusions of the manuscript, but all underlying data and plots were remade.*

**Figure 1**

**General: how many larvae were tested?**

*We aimed to have approximately 100 L1s seeded in each well that corresponded to one of four technical replicates, for a total of approximately 400 worms scored per biological replicate. We have updated the Methods section to clarify.*

**Line 654-660: It is really hard for me to see lines in that figure, maybe they are completely over plotted, but then mention that.**

*Indeed, the dose-response curves for the different strains tested overlap with each other and fall into an N2-like sensitive group and a more resistant group. We tried to address this issue in the main text (lines 132-134 of the original version), but have reiterated this point in the figure description as well.*

**Line 663: was this corrected for multiple testing?**

*The p-values presented were not corrected for the multiple comparisons that were made. The multiple testing adjusted p-value for the DL238 - N2 comparison is 0.087 and the EG4725 comparison is 0.026, using Tukey’s Honest Significant Difference method.*

**Line 664-667: initially looking at the figures and the legend, this confused me, can you mention it is another experiment.**

*We have made this change.*

**Line 668: should be ‘shown in B’)**

*We have made this change.*

**Figure 5**

**General (B): what do the vertical lines indicate? Which of the glct genes?**

**Line 722: Please state what Watterson’s theta indicates.**

*We added these details to the Figure 5 caption:*

*The genetic diversity as measured by Watterson’s theta (Θw) for the genomic regions that contain the six glucuronosyl transferase-like genes in C. elegans is shown. Each vertical line marks the position of a glct gene, from left to right on chromosome I: glct-4, glct-1, glct-3 (pink), glct-2, and glct-5; and chromosome IV: glct-6. Each dot represents a 10,000 bp genomic region and is colored red if the Θw value is greater than the 99th quantile of values across the chromosome.*

**Figure S4**

**General (A): can a strain be represented multiple times? It seems that having a stop, for example, often coincides with Hap:3?**

*Yes, that is correct - a strain can be represented multiple times, but only when that strain contains a variant with a high predicted effect on gene function. In the Figure S4 caption of the submitted manuscript, we included this sentence to indicate this possibility:*

*“If a variant with a high predicted effect on gene function was identified, we plotted it separately.”*

*However, we understand the reviewer’s confusion and have added a sentence to explicitly state that a strain can be represented twice:*

*“If a variant with a high predicted effect on gene function was identified, we plotted it separately. Therefore, a strain can be represented twice if it contains a variant with a high predicted effect on gene function.”*

**Reviewer #3:**

**The authors describe a study to map propionate sensitivity using the C. elegans model system. They take a GWAS approach using a set of 133 wild strains and identified several QTL influencing this trait. For one QTL, they validate a candidate gene, while for a second QTL, validation failed. Overall, this is nice study showing how an animal model can help gain insights into human diseases that are difficult to study. I appreciated the open and thorough discussion of the negative results for the QTL on chromosome V. I do have some concerns and comments, that I detail below and hope will improve the manuscript.**

**1) There are several places where the experimental procedures or data analysis is not explained fully. For example, it would be useful to know the number of worms assayed on each plate and to have an explanation of what constitutes a technical versus biological replicate in this experiment. Maybe I am missing something but I am not sure what a “technical” replicate is for a study quantifying survival of a group of individuals. In addition, I think it would be appropriate to briefly describe the statistical model that is fit by the SKAT approach. There are many different types of gene-based association approaches and it would be helpful to have a bit more information about the approach they have taken. Another example is in Figure 2B, where they refer to the Eigen-decomposition significance threshold but don’t explain this threshold in the Methods.**

*We apologize for any confusion in the methods regarding replicates, another reviewer asked for additional details as well. We have modified the Methods section to explain the following details:*

*Details of propionate assay:*

*A biological replicate is an independent growth of a strain. For each biological replicate, strains were chunked from a starved plate, grown for two generations, bleached, synchronized as L1s in M9, and plated out on agar plates with propionate. For each technical replicate, approximately 100 arrested L1s were transferred to an individual well of a 48-well plate. Because we had a high level of replication, we were not able to complete the entire GWA phenotyping in one experiment and had to phenotype the strains in three separate sets. For each set, we included six reference strains to verify that the batch conditions did not change drastically.*

*SKAT model:*

*We updated the methods section to reference the original manuscript that described the model used in our analysis.*

*In parallel to marker-based GWA mappings, we performed a sequence kernel association test (SKAT), which is implemented in the RVtests software package using SKAT [34, 50].*

*We set the maximum allele frequency for SKAT to 50% using the --freqUpper from flag cegwas2-nf, and the minimum number of strains to share a variant to two using the --minburden flag. The model used for burden mapping was set with the following flag --kernel skat when executing rvtests. This flag implements the burden test that is represented in equation 1 of Wu et al. 2011 [34]. We used the weights suggested by Wu et al. 2011 when running the mapping (beta1 = 1, and beta2 = 25), which adds more weight to rare variants when testing a gene’s variants for association with propionate responses.*

*Eigen-decomposition:*

*On lines 383-390 of the original manuscript, we explained how we determined the eigen threshold. We note that reference 49 (Li and Ji 2005) was in the wrong place and have updated the manuscript accordingly.*

*Lines 383-390: This genotype data set consisted of 59,241 markers that were used to generate the realized additive kinship matrix using the A.mat function in the rrBLUP R package [48]. These markers were also used for genome-wide mappings. However, because these markers still have substantial LD within this genotype set, we performed eigen decomposition of the correlation matrix of the genotype matrix using eigs_sym function in Rspectra package ~~[49]~~ (https://github.com/yixuan/RSpectra). The correlation matrix was generated using the cor function in the correlateR R package (https://github.com/AEBilgrau/correlateR). We set any eigenvalue greater than one from this analysis to one and summed all of the eigenvalues to obtain 772 independent tests within this genotype matrix [49].*

**2) The assay focused on L1 stage survival. How well does this model the human disease? Did the authors consider using survival to (or at) another stage? Some justification of this experimental decision would be helpful.**

*We thank the reviewer's insightful suggestions. In most cases of propionic acidemia, the disease symptoms present within a few days after birth; in less common cases, the signs and symptoms of propionic acidemia appear during childhood and may come and go over time. In C. elegans, we found that propionate has a stronger effect on L1 larvae than later stages. For example, L1s that survive 100 mM propionate treatment will continue to grow to adulthood.  High concentrations of propionate do not affect C. elegans hatching rate, which makes using this phenotype difficult for GWA mapping.*

**3) The presented data are all in proportions. Was the data analyzed as proportions or was a statistical model using a binomial distribution used? This wasn’t clear to me in the Methods. If the raw proportions were used, is the starting number always the same (meaning there is the same amount of uncertainty for all estimates?) In some cases, the median and mean look to be pretty different. What does the distribution of the data and the residuals look like? I would like to see a more in depth discussion of these issues and a justification for the appropriateness of the statistical model for their data.**

*The original data was analyzed as proportions (as shown in supplemental Figure 2B). Both the starting and survived worms were scored separately, which yields a relatively reliable survival rate. However, counting errors prior to and after propionate treatment contribute the most to the variability. This lack of quantitative detail is consistent across all assays, strains, replicates, and conditions, so we believe that it has a small effect on our results. The validated effect by NIL and replacement strains significantly tempered our concerns.*

*The median and means are different between the assays because the underlying data are different. In Figure 2, we are plotting processed data (after outlier removal and assay regression). In Figure S2, we are showing raw data.*

**4) Determination of significance: The authors chose to use a Bonferroni threshold. I would advocate for a false discovery approach instead since Bonferroni is already known to be overly conservative and they are not highly powered with just 133 strains so the risk of type II errors is high.**

*We agree that a less strict threshold would be more appropriate than the Bonferroni threshold used mappings. It is for this reason that we incorporated the eigen threshold for the single-marker mapping, which accounts for correlation among the markers tested for association with strain propionate responses.*

*Regarding the significance threshold for burden mapping, we defined the significance threshold using the Bonferroni method as the reviewer noticed. We have included a permutation-based p-value, which was generated when running the mapping software, in Supplemental Data 6. We did not discuss these results because genes did pass the Bonferroni threshold, which enabled us to perform follow-up experiments on these regions. We view these mapping experiments as discovery tools. The follow-up validation experiments by NILs and allele-replacement strains are the method to determine the role of genes and specific variants in a trait of interest. We do not use mapping data alone.*

**5) The power analysis indicates the number of replicates needed to detect a phenotypic pairwise difference between 2 strains (via a single t-test). This analysis does not indicate the power of the GWAS, which will depend on the effect size of the locus, the heritability of the trait overall (how many other loci contribute), and the number of strains, etc. To avoid any confusion for the reader, I think the authors should state this very clearly so the impression is not given that the analysis indicates the power of the GWAS.**

*We agree with the reviewer that our analysis was only intended to determine the number of replicates required to accurately assess a strain’s phenotype. To prevent any confusion for the reader, we modified the “Power analysis” methods section in the following way (bold text denotes additional phrasing for clarity):*

*To determine the number of replicate measures needed to accurately assess a strain’s phenotype, we measured L1 survival of the DL238 strain after exposure to 100 mM propionic acid in 40 replicates. The 40 replicates consisted of eight technical replicates across five independent preparations of agar plates with propionate. For a range of mean differences (0.01 to 1, in increments of 0.01), we subsampled two to eight replicates for each of the five plate preparations 100 times and calculated the standard deviation of L1 survival for the subsamples. To calculate the power to detect a difference across a range of replicates and mean differences, we used the power.t.test function in the pwr R package with the following parameters - n = number of subsampled replicates, delta = (0.01 to 1, in increments of 0.01), sd = mean of the standard deviation subsamples, sig.level = 0.00001, alternative = “two.sided”, type = “two.sample”. With four technical replicates across five independent plate preparations we were able to detect a 20% difference in means 80% of the time. We note that this analysis does not determine the power to detect QTL via GWA mapping, which will depend on the effect size of a given locus, the number of strains analyzed, the overall trait heritability, interactions among contributing loci, and other factors.*

**6) Patterns of LD: Given the LD patterns observed in these strains, I expected the confidence intervals for the QTLs to be determined by how far strong LD extends, rather than a constant SNP number (100 SNPs). Perhaps the authors could provide a justification for this CI? For the gene-based test, I also think it might be valuable to consider a confidence interval based on LD. From Figure 4a, it looks like LD at least has the potential to drive false signals.**

*We agree with the reviewer’s point that the constant +/- 100 SNPs does not take LD into account. Previously, we simulated a QTL at approximately 15,000 positions in the genome and found that +/- 50 SNPs surrounding a peak QTL marker accurately identified the simulated locus (ref. 26 from the manuscript). However, we have not updated this analysis with our larger strain and marker set, but arbitrarily doubled the size of the interval by 100 SNPs. Of course, we agree that this is not a confidence interval at all and have modified the text accordingly to “region of interest”.*

*With regards to the LD-based confidence interval for burden mapping, only one gene on chromosome I was significantly associated with propionate responses (glct-3) and the LD structure surrounding this locus (shown in Figure S4b) does not suggest that LD is driving spurious signal in this region. We note that three SNPs above -log10(p)=3 presented in Figure 4a (other than those in glct-3) are not in LD with causal Gly16* variant, as indicated in Figure S4b. Furthermore, as the reviewer suggested, the LD structure in the 250 kb surrounding glct-3 strongly implicates variation in this gene as contributing to propionate responses in the C. elegans population, which we went on to experimentally validate.*

**7) I commend the authors for including a frank discussion of the lack of validation for the QTL on chromosome V. Have the authors considered whether the strains chosen for the validation might be driving the lack of result? Given this is a quantitative trait, there are presumably many variants in the genome that influence this trait and the uncertainty associated with localizing a single causative marker, perhaps the two strains chosen did not harbor the causative allele at this QTL.**

*We agree with the reviewer’s conclusion that we might have chosen incorrect strains to validate the chromosome V QTL. However, we were limited by the known incompatibilities in the C. elegans to generate near-isogenic lines, the strain’s phenotypes in the presence of propionate, and the haplotype at the QTL position. We added some of these explanations to the Results section.*

**8) The major motivation for the paper is presented as a way to understand human disease but there is not too much discussion of this point or much background given for other studies focused on identifying modifier loci. The authors might consider expanding these points. Or, if not, they might consider whether a combined Results & Discussion section might serve the paper better.**

*We thank the reviewer for this suggestion. After significant editing, we added more detail to the Introduction and Discussion that, we believe, addresses this point without combining the Results and Discussion.*

**9) Figures:**

**In Figure 1B, I think there should be 12 lines based on what I think is being plotted but I only see maybe 6.**

*Reviewer two raised the same over-plotting issue that we addressed above.*

**In Figure 1E, it would be useful to also plot estimated standard errors. Often for heritability estimates, these can be obtained using a jackknife approach. There is also an error in the legend for 1E – I believe they intended to refer to 1B not 1A.**

*Reviewer two was also curious about why we did show estimate errors for the complete dose response experiment. We have updated the figure to incorporate this information. We have updated the wrong reference in the figure caption.*

**In Figure 2A, if it is possible to plot the raw data points with means and SEs overlaid, I always prefer that to a dynamite plot. The color choices in B are much to close. A lighter pink paired with a darker red might work but 2 more divergent colors would be better.**

*In the original manuscript, we displayed the raw data in Figure S2c with medians overlaid. We have updated this figure to show the means and standard deviations for each strain. We displayed this in the supplemental figures because we think it might be too busy for the main display figures. We hope that this change makes the conclusion clear.*
